# Tumor cell-based vaccine contributes to local tumor irradiation by eliciting a tumor model-dependent systemic immune response

**DOI:** 10.3389/fimmu.2022.974912

**Published:** 2022-09-05

**Authors:** Tinkara Remic, Gregor Sersa, Kristina Levpuscek, Ursa Lampreht Tratar, Katja Ursic Valentinuzzi, Andrej Cör, Urska Kamensek

**Affiliations:** ^1^ Department of Experimental Oncology, Institute of Oncology Ljubljana, Ljubljana, Slovenia; ^2^ Faculty of Medicine, University of Ljubljana, Ljubljana, Slovenia; ^3^ Faculty of Health Sciences, University of Ljubljana, Ljubljana, Slovenia; ^4^ Veterinary Faculty, University of Ljubljana, Ljubljana, Slovenia; ^5^ Biotechnical Faculty, University of Ljubljana, Ljubljana, Slovenia; ^6^ Department of Research, Valdoltra Orthopaedic Hospital, Ankaran, Slovenia; ^7^ Faculty of Education, University of Primorska, Koper, Slovenia

**Keywords:** tumor cell-based vaccine, adjuvant gene electrotransfer, interleukin 12, ionizing irradiation, tumor-infiltrating immune cells, macrophages, effector lymphocytes, regulatory T cells

## Abstract

Multimodal treatment approaches, such as radio-immunotherapy, necessitate regimen optimization and the investigation of the interactions of different modalities. The aim of this study was two-fold. Firstly, to select the most effective combination of irradiation and the previously developed tumor cell-based vaccine and then to provide insight into the immune response to the selected combinatorial treatment. The study was performed in immunologically different murine tumor models: B16F10 melanoma and CT26 colorectal carcinoma. The most effective combinatorial treatment was selected by comparing three different IR regimens and three different vaccination regimens. We determined the local immune response by investigating immune cell infiltration at the vaccination site and in tumors. Lastly, we determined the systemic immune response by investigating the amount of tumor-specific effector lymphocytes in draining lymph nodes. The selected most effective combinatorial treatment was 5× 5 Gy in combination with concomitant single-dose vaccination (B16F10) or with concomitant multi-dose vaccination (CT26). The combinatorial treatment successfully elicited a local immune response at the vaccination site and in tumors in both tumor models. It also resulted in the highest amount of tumor-specific effector lymphocytes in draining lymph nodes in the B16F10, but not in the CT26 tumor-bearing mice. However, the amount of tumor-specific effector lymphocytes was intrinsically higher in the CT26 than in the B16F10 tumor model. Upon the selection of the most effective combinatorial treatment, we demonstrated that the vaccine elicits an immune response and contributes to the antitumor efficacy of tumor irradiation. However, this interaction is multi-faceted and appears to be dependent on the tumor immunogenicity.

## 1 Introduction

In recent years, it has become clear that a multimodal approach is a necessity for treatment of most tumors. One such multimodal approach is radio-immunotherapy (1). Ionizing radiation (IR) is one of key cancer treatment modalities. It is largely used for its ablative effects, namely genome instability driven cell death, while its immunological properties remain a controversial topic among scientists (2, 3). Aside from apoptosis, IR can lead to immunogenic cell death and consequently to activation of dendritic and T cells (1, 4, 5). Another immunological property of IR is immunogenic modulation, whereby IR causes phenotypical changes such as increase in tumor antigen presentation and diversity (6, 7). Reports of IR alone invoking a substantial immune response (abscopal effect) are rare; however, immunotherapy can and has been used to boost IR (1, 8).

The most extensively researched and currently most effective radio-immunotherapy is the combination of checkpoint inhibitors and IR (1, 8). Nonetheless, non-responding patients remain and it is important to investigate alternatives such as the combination of therapeutic vaccination and IR (9). Tumor cell-based vaccines, a type of therapeutic vaccines, use tumor cells taken from patients as the source of tumor antigens (10–12). These tumor cells are commonly genetically modified *ex vivo* and then inactivated with high-dose IR or lysed (10, 11). We have previously developed an effective alternative tumor cell-based vaccine, which consists of IR-killed non-viable tumor cells and plasmid DNA encoding murine interleukin-12 (IL-12) (13). The non-viable tumor cells in our vaccine were used as the source of tumor antigens and were not genetically modified *ex vivo* unlike in vaccines such as Algenpantucel-L or GVAX (11, 14). While, *in vivo* gene electrotransfer (GET) of the IL-12 plasmid contained within our vaccine was administrated as the immunological adjuvant (13).

GET is a non-viral form of gene therapy that can be used *in situ* (15–21). By applying set of electrical pulses we transiently permeabilize the targeted tissue, thus enabling the transfer of locally injected target plasmid DNA into the cells (15–21). Common immunological adjuvants are cytokines and chemokines such as granulocyte-macrophage colony stimulating factor, interleukin-2 and IL-12 (22). The latter is a proinflammatory cytokine that enhances effector T and NK cell maturation and their cytotoxicity as well as recruits macrophages and enhances the immune response of helper T cells (Th cells) (23, 24). IL-12 GET has been tested in numerous pre-clinical and clinical studies as local intratumoral treatment or as an adjuvant to vaccination or other therapeutic approaches such as electrochemotherapy (15–21).

In this study, we investigated some of the typical challenges of combining radio- and immunotherapy such as IR dose regimens, vaccination regimens and timing of the combinatorial treatment. Our aim was to select the most effective combination of IR and the previously developed tumor cell-based vaccine (13) as well as provide insight into the therapy-induced immune response. The study was performed in immunologically different murine tumor models: B16F10 melanoma and CT26 colorectal carcinoma (25, 26). We began with IR regimen selection followed by vaccine regimen selection and, lastly, we investigated the immune response at the site of vaccination, tumor and draining lymph nodes.

## 2 Materials and methods

### 2.1 Cell cultures

Murine cell lines B16F10 melanoma (CRL-6475, American Tissue Cell Culture (ATCC), Manassas, USA) and CT26 colon carcinoma (CRL-2638, ATCC) were cultured at standard conditions: 37°C, humidified atmosphere containing 5% CO_2_, in Advanced Modified Eagles Medium (A-MEM, Thermo Fisher Scientific, Waltham, USA) and Advanced Roswell Park Memorial Institute medium (A-RPMI, Thermo Fisher Scientific), respectively. Culture media were supplemented with 5% fetal bovine serum (FBS; Thermo Fisher Scientific), 10 mM L-glutamine (Thermo Fisher Scientific), 50 mg/mL gentamicin (Krka, Novo mesto, Slovenia) and 100 U/mL penicillin (Sandoz International GmbH, Holzkirchen, Germany). Both cell lines were used within 10 passages and were found negative during continuous testing every 3 months for mycoplasma with MycoAlert™ PLUS Mycoplasma Detection kit (Lonza, Basel, Switzerland).

### 2.2 Plasmid DNA

Plasmid DNA pORF-mIL-12-ORT encoding murine IL-12 without an antibiotic resistance gene (27) was isolated using the EndoFree Plasmid Mega Kit (Qiagen, Hilden, Germany). Plasmid purity and the concentration were determined using the Epoch Microplate Spectrophotometer, Take3™ Micro-Volume Plate (BioTek, Bad Friedrichshall, Germany). After confirming plasmid identity with restriction enzyme analysis on a 1% agarose electrophoretic gel, aliquots of 50 µg of IL-12 plasmid were vacuum dried using the Concentrator plus (60°C, VA-Q program, Eppendorf, Hamburg, Germany).

### 2.3 Vaccine preparation

B16F10 and CT26 vaccines containing non-viable tumor cells and IL-12 plasmid were prepared as described previously (13). Briefly, cells were irradiated at a dose rate of 1.728 Gy/min using the Darpac 3300 X ray unit (Gulmay Medical Ltd., Byfleet, UK) operating at 200 kV and 9.2 mA with a 0.55 mm Cu and 1.8 mm Al filtration. B16F10 cells were exposed to 3 fractions of 5 Gy and a single lethal fraction of 30 Gy, while the more radioresistant CT26 cells were exposed to 3 fractions of 5 Gy and 2 fractions of 30 Gy. Harvested non-viable B16F10 or CT26 tumor cells were resuspended in concentrated harvested media to a final protein concentration of 10 mg/mL, which was determined with the Pierce™ bicinchoninic acid (BCA) Protein Assay Kit (Thermo Fisher Scientific). Finally, 50 µg of IL-12 plasmid were added to 100 µL of 1× 10^6^ non-viable tumor cells to make 1 unit of B16-F10 and CT26 vaccines (1 mg/U).

### 2.4 Animals

Female 6-8 week-old C57BL/6NCrl and BALB/cAnNCrl mice (Charles Rivers, Calco, Italy) were maintained in an animal colony in a 12h light/dark cycle under specific pathogen-free conditions at constant room temperature and humidity. Food and water were provided *ad libitum*. Animals were subjected to a quarantine and adaptation period of 2 weeks before the *in vivo* experiments. All experimental procedures were performed in accordance with the EU directive (2010/63/EU) and with the guidelines of the Ministry of Agriculture, Forestry, and Food of the Republic of Slovenia (permission no. U34401–1/2015/43 and U34401-35/2020/8).

### 2.5 Tumor induction

B16F10 and CT26 tumors were induced with a subcutaneous injection of 0.5× 10^6^ viable B16-F10 or CT26 tumor cells into the lower backs of respective syngeneic mice. The general well-being of mice was monitored by their weight, ease of movement and behavior.

### 2.6 Tumor treatment

#### 2.6.1 Tumor irradiation

Induced tumors were irradiated at a dose rate of 1.92 Gy/min using the Darpac 3300 X ray unit (Gulmay Medical Ltd.) operating at 200 kV and 9.2 mA with a 0.55 mm Cu and 1.8 mm Al filtration. Mice were restrained in special lead tubes with fixed apertures during irradiation and were put sideways on a stand at a fixed distance from the X ray machine head. The tumors were exposed to ½ of the dose on each side to equalize dose distribution.

#### 2.6.2 Vaccination

Vaccination was performed distantly from the tumor as described previously (13). Briefly, a unit of B16F10 or CT26 vaccine (1 mg/U) was injected subcutaneously in the upper back of mice. A contact hexagonal multi-electrode array with the central pin (MEA, Iskra Medical, Ljubljana, Slovenia) connected to a Cliniporator (IGEA s.r.l., Carpi, Italy) was positioned to encompass the injected vaccine containing IL-12 plasmid. Adjuvant GET was performed by administering 24 electrical low voltage pulses with an amplitude-over-distance ratio 170 V/cm, duration of 150 ms and frequency of 5.64 Hz.

#### 2.6.3 Treatment regimens

##### 2.6.3.1 Irradiation regimens

**Figure 1:**
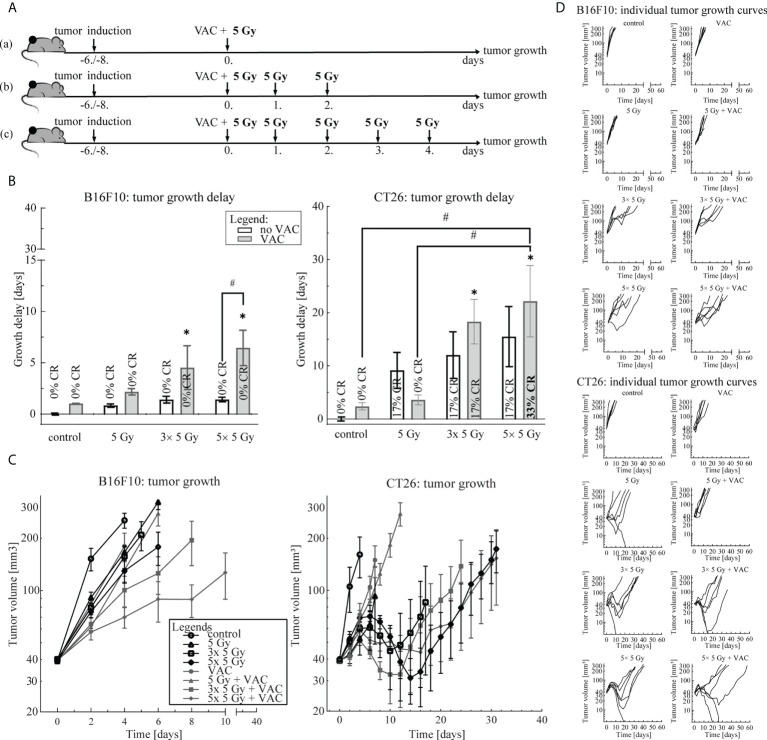
Irradiation regimen selection. **(A)** Treatment timelines: (a) single-dose vaccination combined with 1× 5 Gy, (b) single-dose vaccination combined with 3× 5 Gy and (c) single-dose vaccination combined with 5× 5 Gy. **(B)** Tumor growth delay in the B16F10 (left) and the CT26 (right) tumor model. The legend applies to both graphs. **(C)** Tumor growth over time in the B16F10 (left) and the CT26 tumor model. The legend applies to both graphs. **(D)** Individual growth curves for each tumor of each treatment (control) group in the B16F10 (top) and the CT26 (bottom) tumor model. Legend: *p < 0.05 versus control group; ^#^p < 0.05 between the annotated treatment groups; CR, complete response; VAC, single-dose vaccination.

Three irradiation regimens: (a) 1× 5 Gy, (b) 3× 5 Gy and (c) 5× 5 Gy, in combination with single-dose vaccination were compared (**Figure 1**). Mice were randomly divided into treatment and control groups (n = 6 mice per group) when tumor size reached 35-40 mm^3^ (Day 0). Tumors were irradiated with a fraction per day. Mice in control groups did not receive any treatment. The most effective irradiation regimen was chosen for further experiments.

##### 2.6.3.2 Vaccination regimens

**Figure 2:**
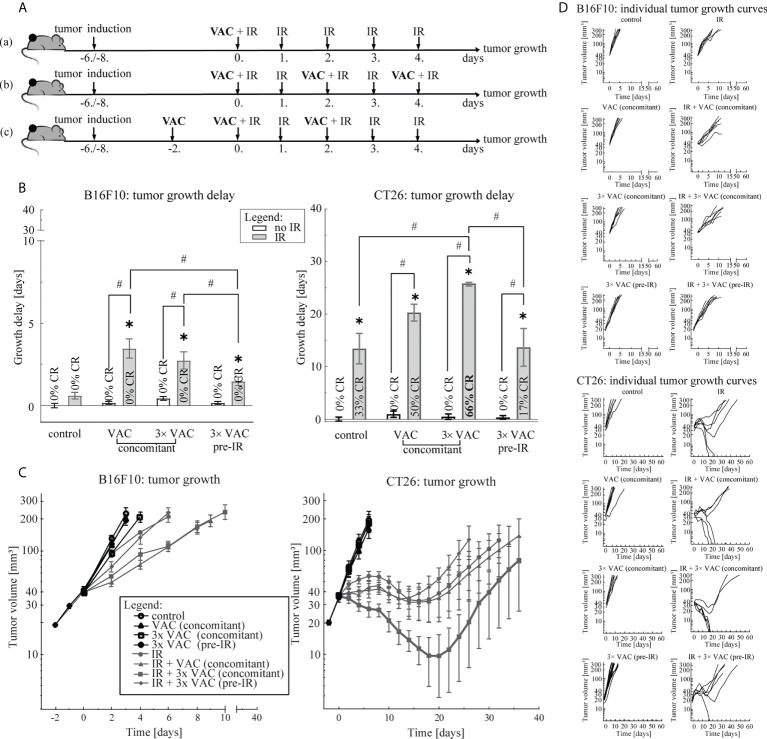
Vaccination regimen selection. **(A)** Treatment timelines: (a) concomitant single-dose vaccination combined with IR, (b) concomitant multi-dose vaccination combined with IR and (c) pre-IR vaccination combined with IR. **(B)** Tumor growth delay in the B16F10 (left) and the CT26 (right) tumor model. The legend applies to both graphs. **(C)** Tumor growth over time in the B16F10 (left) and in the CT26 (right) tumor model. The legend applies to both graphs. **(D)** Individual growth curves for each tumor of each treatment (control) group in the B16F10 (top) and the CT26 (bottom) tumor model. Legend: *p < 0.05 versus control group; ^#^p < 0.05 between the annotated treatment groups; CR, complete response; VAC, single-dose vaccination; IR, selected 5× 5 Gy IR regimen.

Three vaccination regimens: (a) concomitant single-dose, (b) concomitant multi-dose and (c) pre-IR multi-dose vaccination, in combination with the selected tumor irradiation regimen were compared (**Figure 2**). Mice were randomly divided into treatment and control groups (n = 6 mice per group) when tumor size reached 18-23 mm^3^ (day -2). Concomitant single- and multi-dose vaccination started on day 0, while pre-IR multi-dose vaccination started on day -2. For multi-dose vaccinations, each vaccination dose (1 mg/U) was administered every 2 days for a total number of three vaccination doses. Tumor IR (5× 5 Gy) started on day 0 with a fraction per day. Mice in control groups did not receive any treatment. The most effective vaccination regimen was chosen for histological and FluoroSpot analysis of combinatorial treatment.

### 2.7 Tumor growth follow up

Every second day three orthogonal diameters of the tumor (a, b, c) were measured using a Vernier Caliper and tumor volumes were calculated using the following formula:


$$V\;\;=\;\;\frac{a\;\times \;b\;\times \;c\;\times \;\pi }{6}$$

The doubling time (DT) of a tumor was determined as the time, when the tumor reached twice the starting volume, i.e. approximately 70-80 mm^3^. Tumor growth delay (GD) was then calculated by subtracting the average DT of untreated tumors from DT of treated tumors using the following formula:


$$GD\;=\;\bar{DT_{tr}}\;-\;\bar{DT_{untr}}\;$$

Mice were sacrificed using a CO_2_ chamber or *via* cervical dislocation when they reached the humane end-point: tumor size of 300 mm^3^ or before harvesting tissues for further analysis. A complete response was defined as the absence of a detectable tumor for 100 days.

### 2.8 Histological analysis

Skin at the vaccination site and tumors in B16F10 and CT26 tumor-bearing mice (n = 3 mice per group) were aseptically harvested 6 days after the start of the selected combinatorial or control treatment. Zinc-fixed paraffin-embedded samples were cut in 2-5 µm sections and stained with hematoxylin and eosin (HE) or immunohistochemically. Immunohistochemical (IHC) staining was performed using the Rabbit Specific HRP/AEC IHC Detection Kit-Micro-polymer (ab236468; Abcam, Cambridge, UK) following the manufacturer protocol. The primary CD68 polyclonal antibody (PA5-78996; Invitrogen, Thermo Fisher Scientific) at dilutions 1:1000 for tumor samples and 1:1250 for skin samples was used, while the primary Anti-Granzyme B antibody (ab4059; Abcam, Cambridge, UK) at 1:1000 dilution and the primary Foxp3 Antibody (700914; Thermo Fisher Scientific) at 1:1200 dilution were used for both tissue samples. Hematoxylin was used as a counterstain.

Samples were observed at 40× magnification using a BX-51 microscope (Olympus, Düsseldorf, Germany). For immunohistochemical analysis of tumor-infiltrating immune cells, 10 images per sample were taken using the microscope-connected camera (DP72 CCD, Olympus). The average numbers of macrophages (CD68^+^), effector lymphocytes (GrB^+^) and regulatory T cells (FoxP3^+^) were assessed in a blind fashion by 3 examiners (28) and fold change was calculated using the following formula:


$$fold\;change\;=\;\frac{average\;number\;of\;cells\;of\;interest\;in\;the\;treatment\;group}{average\;number\;of\;cells\;of\;interest\;in\;the\;control\;group}$$

Data was presented as the fold change relative to control samples for each tumor model.

### 2.9 FluoroSpot analysis

#### 2.9.1 Lymphocyte isolation

Peripheral skin-draining lymph nodes (inguinal, brachial and axillar) were aseptically harvested from B16-F10 and CT26 tumor-bearing mice (n = 3 mice per group) 6 days after the start of the selected combinatorial or control treatment. The lymph nodes were sheared using a 50 μm sterile strainer (Sysmex, Norderstedt, Germany) and the lymphocytes were suspended in 10 mL of cooled phosphate buffer solution (PBS, 4°C). The cell suspension was centrifuged at 470g, 4°C for 5 min and the pellet was washed with cooled PBS (4°C). After an additional centrifugation at 470g, 4°C for 5 min the pellet was resuspended in 1 mL of cooled Serum-Free Freezing Media (4°C, Biological Industries, Beit HaEmek, Israel). Isolated lymphocytes were stored at -80°C for future FluoroSpot analysis.

#### 2.9.2 Sample thawing

The thawing process began with a short incubation of cryovials at 37°C. Under sterile conditions, 1 mL of warm RPMI HEPES media (37°C, Sigma-Aldrich, St. Louis, USA) was slowly added to the cell suspension, which was later transferred into 8 mL of warm RPMI HEPES media. To prevent cell clumping, the cell suspension was centrifuged at 500g for 10 min at room temperature. The pellet was resuspended in 2 mL of Hank’s Balanced Solution with calcium and magnesium (HBSS, Gibco, Thermo Fisher Scientific). DNaze I (2 U/mL, Invitrogen, Thermo Fisher Scientific) was added and the suspension was incubated with shaking (200 rpm) at 37°C for 45 min. Warm RPMI HEPES was added until the concentration reached 2× 10^6^ cells/mL. Cells were allowed to recover overnight at standard cell culture conditions.

#### 2.9.3 FluoroSpot and data analysis

To determine the presence of tumor specific immune cells, Dual-Color FluoroSpot Mouse IFNγ/GrB kit (R&D Systems, Minneapolis, USA) was used according to manufacturer instructions. Isolated immune cells (1× 10^4^ per well) were stimulated with tumor cells in a 10:1 ratio. The green (GrB^+^ spots) and red (IFNγ^+^ spots) fluorescent spots were captured at 488 nm and 588 nm, respectively, using the Zeiss SteREO Lumar.V12 equipped with Zeiss AxioCam (Zeiss, Oberkochen, Germany). The images were processed in ImageJ (29, 30) and the spots were counted manually. The fold change was calculated using the following formula:


$$fold\;change\;=\;\frac{number\;of\;stimulated\;GrB^{+}\;/\;IFN\gamma^{+}\;immune\;cells\;in\;the\;treatment\;group\;}{number\;of\;stimulated\;GrB^{+}\;/\;IFN\gamma^{+}\;immune\;cells\;in\;the\;control\;group}$$

The data was presented as the fold change relative to stimulated (tumor specific) GrB^+^/IFNγ^+^ immune cells in the control group.

### 2.10 Statistical analysis

Data analysis was performed using GraphPad Prism version 8.1.2. (GraphPad Software, US). The Shapiro-Wilk test was used to test the normal data distribution. Data were presented as the mean ± standard error (SE) and a One-way ANOVA followed by the Holm-Sidak test for multiple comparisons was performed for the determination of significant differences (p< 0.05) between experimental groups. Complete responses were not included in the statistical analysis of GD. To include complete responses, data was further analyzed by comparing the probability of reaching a doubling tumor volume using a log-rank test for trend. The event, marked as 1, was a tumor reaching double the starting size (doubling volume), while a censored event, marked as 0, was a tumor reaching a complete response. A log-rank (Mantel-cox) test was then used to determine statistical significance (p< 0.05) between treatment groups. Student’s T test was used to determine significant differences (p< 0.05) between the individual tumor growth curves by comparing the tumor volumes of different treatment groups at individual time points. Student’s T test was also used to determine significant differences (p< 0.05) between the two tumor models.

## 3 Results

### 3.1 Irradiation regimen selection

To select the most effective IR regimen in combination with single-dose vaccination, we compared three IR regimens: (a) 1× 5 Gy, (b) 3× 5 Gy and (c) 5× 5 Gy (**Figure 1A**). We observed a trend of IR dose-dependent antitumor efficacy of the combination therapy in both tumor models (**Figure 1**). In the B16F10 tumor model, single-dose vaccination significantly contributed only to antitumor efficacy of 5× 5 Gy (**Figure 1B**). Whilst a similar trend in tumor growth delay was observed in the CT26 tumor model, the contribution of vaccination to IR was not statistically significant (**Figure 1B**). However, the highest number of complete responses (CR) were observed in combinatorial treatment of single-dose vaccination and 5× 5 Gy (33%) (**Figures 1B, D**). Additionally, IR alone was more effective in the CT26 tumor model than in the B16F10 tumor model (**Figures 1B, C**). Based on these results, we selected the IR regimen of 5× 5 Gy for further studies in both tumor models.

### 3.2 Vaccination regimen selection

To select the most effective vaccination regimen in combination with the selected 5× 5 Gy IR regimen, three vaccination regimens we compared: (a) concomitant single-dose, (b) concomitant multi-dose and (c) pre-IR multi-dose vaccination (**Figure 2A**). In the B16F10 tumor model, no differences between concomitant single- and multi-dose vaccination were observed, although a trend of a greater antitumor efficacy after single-dose vaccination concomitant with IR was observed (**Figure 2**). However, in the CT26 tumor model concomitant multi-dose vaccination and IR led to twice the amount of CR (66% versus 33%) and a statistically significant greater antitumor efficacy than IR alone (**Figure 2B**; **Supplementary Figure 1**), although individual tumor growth curves did not differ significantly at different time points. In both tumor models, the pre-IR multi-dose vaccination was the least effective (**Figure 2**). Since there was no additional benefit, neither in GD nor CR (**Figure 2**), of increasing the amount of vaccination doses in the B16F10 tumor model, we selected the simpler of the two most effective vaccination regimens for further studies, i.e. concomitant single-dose vaccination. In the CT26 tumor model, only concomitant multiple vaccination doses significantly contributed to IR alone and resulted in a greater amount of CR compared to IR alone (**Figure 2**; **Supplementary Figure 1**); thus, the concomitant multiple-dose vaccination regimen was chosen for further studies.

### 3.3 Local immune response at the vaccination site and in the irradiated tumor

To determine the local immune response to the selected combinatorial treatment, the infiltration of macrophages (CD68^+^ cells), effector lymphocytes (GrB^+^ cells), such as effector T and NK cells, and Treg (FoxP3^+^ cells) present at the site of vaccination and in tumors was determined on day 6.

**Figure 3:**
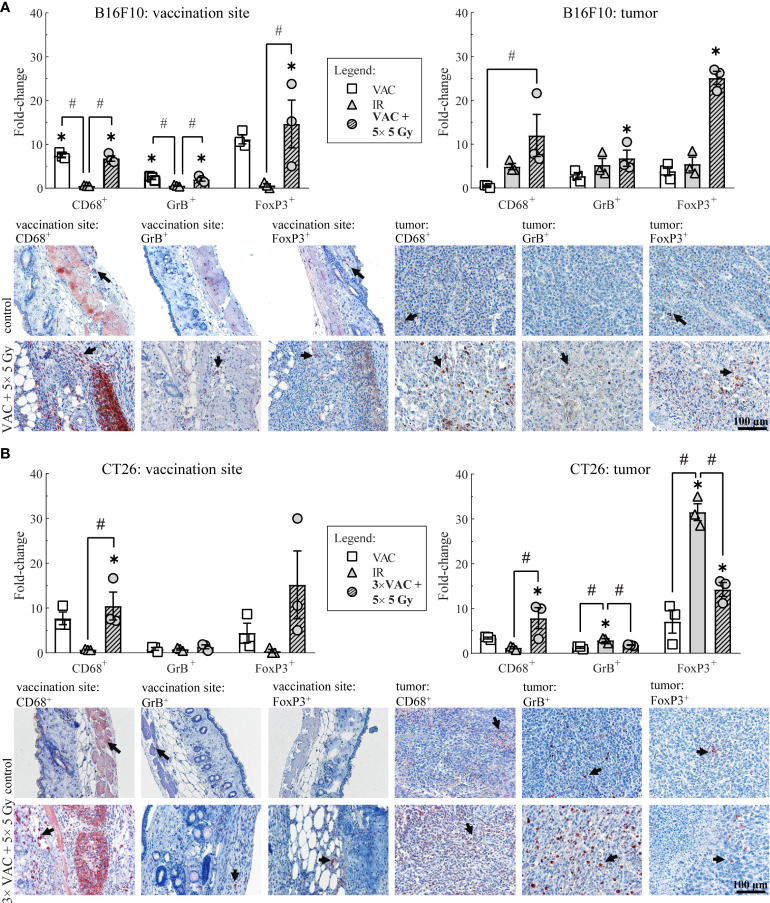
Histological analysis of skin at the site of vaccination and tumors on day 6. **(A)** Immune cell infiltration in the skin at the site of vaccination and in B16F10 tumors. Representative images of the control (untreated mice) and the combinatorial treatment (VAC + 5× 5 Gy) groups are shown underneath the graphs. **(B)** Immune cell infiltration in the skin at the site of vaccination and in CT26 tumors. Representative images of the control (untreated mice) and the combinatorial treatment (VAC + 5× 5 Gy) groups are shown underneath the graphs. Legend: the black arrows in the images indicate examples of cells positive for CD68, GrB or FoxP3 markers; the scale bar for all images is shown in the lower left corner image: 100 µm; *p < 0.05 versus the control group; ^#^p < 0.05 between the annotated treatment groups: VAC, selected concomitant single- and multi-dose vaccinations for B16F10 and CT26 tumor models, respectively; IR, selected 5× 5 Gy IR regimen.

In the B16F10 tumor-bearing mice, the highest infiltration of all above mentioned immune cells at the site of vaccination was detected after vaccination alone and after combinatorial treatment, while in tumors the highest infiltration of immune cells was observed after the combinatorial treatment (**Figure 3A**; **Table 1**). Similarly, in the CT26 tumor-bearing mice, the highest infiltration of macrophages at the site of vaccination was detected after vaccination alone and combinatorial treatment, while in tumors the highest infiltration of macrophages was after combinatorial treatment that was significantly different compared to the control and IR alone (**Figure 3B**; **Table 1**). Interestingly, no changes in infiltration of effector lymphocytes were observed at the site of vaccination, while in tumors the highest infiltration of effector lymphocytes was after IR alone. Lastly, a trend of increased infiltration of Treg cells was observed after combinatorial treatment at the site of vaccination, while in tumors the highest infiltration of Treg cells was determined after IR alone. Although, the infiltration of Tregs in tumors after combinatorial treatment was significantly higher than after control treatment it was also significantly lower than after IR alone.

**Table 1:** The amount of immune cells at the vaccination site and in tumor in both tumor models.

		B16F10 tumor model						CT26 tumor model					
		vaccination site/skin			tumor			vaccination site/skin			tumor		
**CD68^+^**	control	*179,44*	*±*	*45,48*	*2,67*	*±*	*0,51*	*176,89*	*±*	*11,43*	*48,22*	*±*	*14,60*
	VAC	*1370,33*	*±*	*425,37*	*1,11*	*±*	*0,59*	*1382,48*	*±*	*319,12*	*158,78*	*±*	*37,14*
	IR	*106,00*	*±*	*67,28*	*12,58*	*±*	*2,75*	*129,22*	*±*	*19,25*	*64,78*	*±*	*29,56*
	VAC + IR	*1272,00*	*±*	*445,56*	*29,08*	*±*	*8,02*	*1891,33*	*±*	*649,64*	*206,11*	*±*	*36,17*
**GrB^+^**	control	*44,78*	*±*	*8,80*	*0,78*	*±*	*0,29*	*11,56*	*±*	*4,82*	*118,33*	*±*	*39,12*
	VAC	*108,11*	*±*	*32,49*	*2,00*	*±*	*0,84*	*9,74*	*±*	*6,53*	*139,22*	*±*	*20,31*
	IR	*28,17*	*±*	*19,44*	*3,33*	*±*	*1,01*	*8,89*	*±*	*4,44*	*319,44*	*±*	*60,92*
	VAC + IR	*98,00*	*±*	*38,98*	*4,42*	*±*	*0,92*	*14,70*	*±*	*9,20*	*196,44*	*±*	*50,46*
**FoxP3^+^**	control	*8,00*	*±*	*2,27*	*0,78*	*±*	*0,11*	*2,67*	*±*	*2,17*	*17,00*	*±*	*16,00*
	VAC	*84,56*	*±*	*18,60*	*2,78*	*±*	*0,48*	*5,15*	*±*	*2,66*	*46,89*	*±*	*42,07*
	IR	*5,75*	*±*	*8,09*	*4,58*	*±*	*3,41*	*2,67*	*±*	*2,67*	*36,33*	*±*	*7,07*
	VAC + IR	*93,67*	*±*	*23,53*	*19,75*	*±*	*3,71*	*17,41*	*±*	*8,95*	*73,89*	*±*	*56,86*

Data is presented as the arithmetic mean ± standard error.

### 3.4 Tumor specific immune response in draining lymph nodes

**Figure 4:**
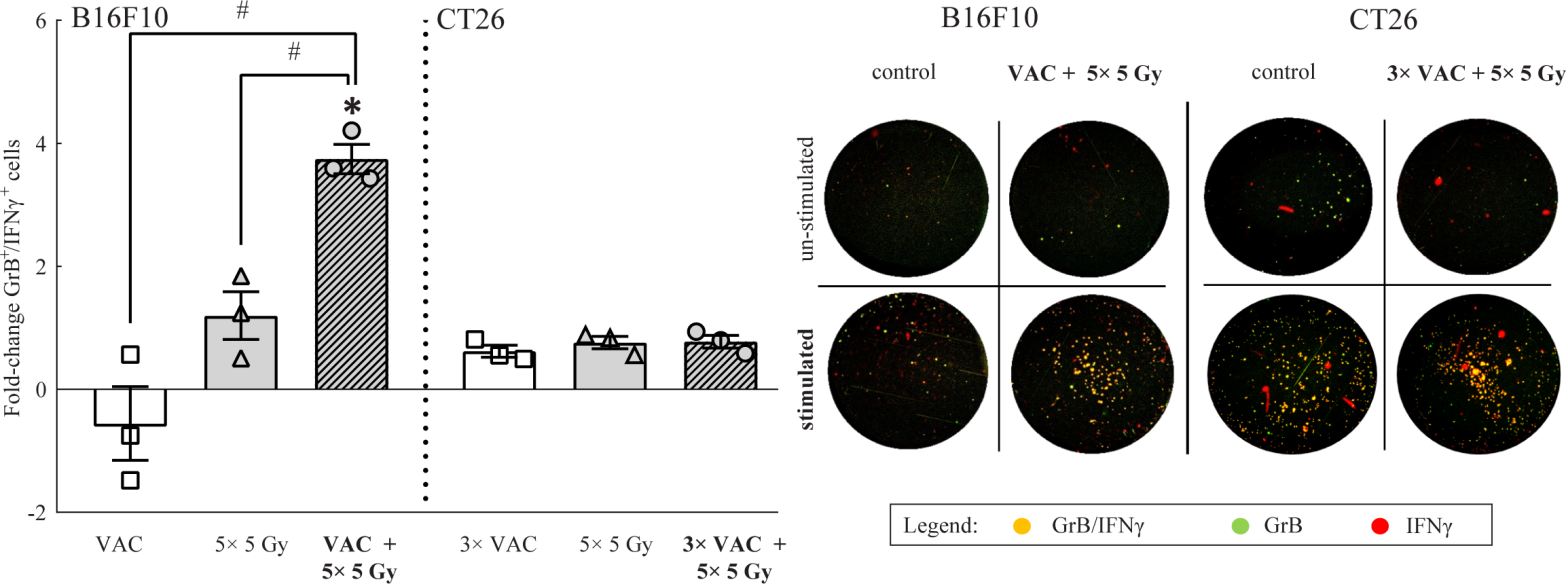
FluoroSpot analysis of tumor specific GrB+/ IFNγ+ immune cells from lymph nodes on day 6. The amount of tumor specific GrB+/ IFNγ+ immune cells from lymph nodes of both tumor-type bearing mice. Representative images of FluoroSpot wells for both tumor models is shown on the right side of the graph. The orange/yellow spots represent double positive immune cells. Legend: fold-change relative to the absolute number of stimulated tumor specific GrB+/ IFNγ+ immune cells of the control group; *p < 0.05 versus the control group; ^#^p < 0.05 between the annotated treatment groups: VAC = selected concomitant single- and multi-dose vaccinations for B16F10 and CT26 tumor models, respectively; IR = selected 5 × 5 Gy IR regimen.

To determine the systemic immune response to the selected combinatorial treatment, tumor-specific GrB^+^/IFNγ^+^ immune cells present in the draining lymph nodes were analyzed on day 6. In B16F10 tumor-bearing mice the highest amount of tumor specific GrB^+^/IFNγ^+^ immune cells was determined in mice receiving combinatorial treatment, while in CT26 tumor-bearing mice, no significant changes were observed in the amount of tumor specific GrB^+^/IFNγ^+^ immune cells regardless of the administered therapy (**Figure 4**). It is worth mentioning that the inherent amount of tumor specific GrB^+^/IFNγ^+^ immune cells in lymph nodes of CT26 tumor bearing mice was significantly higher (987.17 ± 83.88) than in lymph nodes of B16F10 tumor bearing mice model (58.33 ± 28.19) as evident from the representative image of the stimulated control FluoroSpot wells (**Figure 4**).

## 4 Discussion

Multimodal treatment approaches, such as radio-immunotherapy, necessitate regimen optimization and the investigation of the interactions between different modalities. The aim of this study was to select the most effective combination of IR and the previously developed tumor cell-based vaccine (13) and then to provide insight into the immune response to the selected combinatorial treatment.

Our study showed an interdependence of our previously developed vaccine (13) and local tumor IR. By comparing different IR and vaccination regimens, we observed a significant benefit of the final selected combinatorial treatment, which differed based on the tumor model. Namely, the final selected combinatorial treatment was 5× 5 Gy with concomitant single-dose vaccination for B16F10 tumor model and 5× 5 Gy with concomitant multi-dose vaccination for the CT26 tumor model. In this study, we first compared different fractionated IR regimens with a dose of 5 Gy that falls into the reported immunostimulating range of IR doses (4, 6, 31–35). When considering the contribution of vaccination to IR, the effect of vaccination appeared greater when combined with a higher number of IR fractions than with single dose IR. This was statistically significant in the B16 F10 tumor model, where the difference in GD between 5 Gy + VAC and 5 Gy was 1.3 days while the difference in GD between 5× 5 Gy + VAC and 5× 5 Gy was 5 days. This finding coincides with studies that have found IR dose-dependent increase in not only the tumor control but also in the immune response (4, 34, 35). Golden et al. observed a dose-dependent increase in IR-induced immunogenic cell death, which elicits an immune response (4). IR also increases tumor antigen exposure (6, 31, 32). Therefore, it is possible that with increasing number of fractions of 5 Gy, we exposed an increasing amount of tumor cells as targets for the immune response elicited by single-dose vaccination.

During vaccine development we observed that CT26 cells were intrinsically more radioresistant than B16F10 cells *in vitro* due to the higher IR dose needed for vaccine preparation (13). However, we observed the opposite *in vivo*, where the CT26 tumor model was more radiosensitive than the B16F10 tumor model, i.e. 10 days of tumor GD and 17% CR in the CT26 tumor model versus 2 days and 0% CR in the B16F10 tumor model after single 5 Gy irradiation. Therefore, the difference in the intrinsic tumor immunogenicity (25, 26) may also affect the response to the combinatorial treatment as indicated by the greater efficacy of IR alone in the CT26 tumor model compared to the B16F10 tumor model. This resulted in a seemingly ‘lesser’ contribution of vaccination to the final antitumor efficacy in the CT26 tumor model. It has been shown that in the more immunogenic CT26 tumor models ablative therapies have a greater efficacy than in the less immunogenic tumor model B16F10, while the opposite can be said for the contribution of immunotherapy (20).

After comparing different irradiation regimens, we also compared different vaccination regimens. Qiu et al. showed that the immune response is dependent on the number of vaccination doses (36). Three applications of a vaccine resulted in the highest antitumor immune response in the murine lung cancer TC-1 tumor model, while five applications resulted in an immunosuppressive tumor environment (36). In our study, increased benefit of concomitant multiple-dose vaccination was observed only in the CT26 tumor model, while in the B16F10 tumor model, it resulted in comparable antitumor efficacy to concomitant single-dose vaccination. We believe that the observed plateau in the response to vaccination in B16F10 tumor model is due to the limited tumor antigen variety (mutational burden) and exposure (low MHC expression) compared to the CT26 tumor model (25, 26, 37).

Contrary to our expectations and literature, the pre-IR multiple-dose vaccination did not contribute to the antitumor effectiveness of IR in either tumor model. Several studies showed neoadjuvant vaccination, such as IL-2 or dendritic cell infusions, increased antitumor effectiveness of IR and resulted in an increased immune response in murine melanoma B78 tumor model or patients with soft tissue sarcoma (38, 39). Neoadjuvant vaccination with a recombinant Modified Vaccinia Ankara expressing Influenza HA antigen also resulted in increased survival after surgical removal of murine mesothelioma AB1 tumors (40). On the other hand, we observed that vaccination alone or pre-IR multi-dose vaccination in combination with IR were inefficient. This indicates that tumor IR is necessary for the developed vaccine to work. The argument for concomitant vaccination and IR may be three-fold: (1) IR leads to increased tumor antigen presentation (6, 31, 32), (2) it enhances cell stress signals (4, 33, 34), and (3) it delays tumor growth, thus sensitizing the tumor to concomitant vaccination and allowing time for immune response formation.

By investigating immune cell populations in tumors, vaccination sites, and lymph nodes, we further confirmed the necessity of tumor irradiation for the vaccination effects to be apparent in both tumor models. The increase in the infiltration of macrophages at the vaccination site and in tumors was expected due to the non-viable tumor cells present in the vaccine, IL-12 GET, and IR-induced tumor damage (41, 42). Although macrophages have a duplicitous nature (43, 44), we observed the highest infiltration of macrophages in tumors of both tumor models after the most effective combinatorial treatment. This indicates that vaccination successfully elicited the antigen presenting function of macrophages (41). Additionally, doses in the range of 1-10 Gy were found to increase differentiation into proinflammatory macrophages (42, 45, 46). Therefore, tumor IR may have further enhanced the beneficial macrophage response rather than the detrimental anti-inflammatory one (42–46).

Unlike macrophages, effector lymphocytes and Treg cells are generally associated with a positive or negative prognosis, respectively (44). Studies have shown that both vaccination alone and IR alone lead to increased tumor infiltration of both immune cell types (32, 47–49). Concordantly, we observed the highest amount of both immune cell types at the vaccination site and in tumors as well as tumor-specific effector lymphocytes in lymph nodes after combinatorial treatment in the B16F10 tumor model. Because the less immunogenic B16F10 tumor model has a low antigen variety and presentation (25, 26), our vaccine containing most if not all antigens allows for increased antigen presentation through phagocytic immune cells such as macrophages. However, the main tumor site remains ‘hidden’ from the immune system. Thus, we believe that tumor IR, which increases antigen presentation (6, 31, 32), is necessary for the vaccination-induced immune response to spread into the tumor. Additionally, the increased Treg infiltration may have occurred as the side effect of stimulating the immune system. This may lead to immunosuppression; therefore, it might be beneficial to combine this therapy with immune checkpoint inhibitors. On the other hand, the combinatorial treatment was more effective than IR alone despite the increased Treg infiltration, this may suggest that the Treg cells were nonfunctional (50); however, further studies are needed to determine the mechanism behind this immune response.

Unexpectedly, IR alone led to the highest infiltration of both effector lymphocytes, such as effector T and NK cells, and Treg cells in the more immunogenic CT26 tumor model. However, combinatorial treatment led to a significantly lower infiltration of Treg cells in tumors than IR alone. Thus, IR alone may have led to IR-induced immunosuppression (14, 51) in the CT26 tumor model, whereas vaccination potentially reduced it. Namely, our vaccine comprises most CT26 tumor model antigens as well as IL-12 GET (13, 17), which may lead to boosted antigen presentation to lymphocytes. Furthermore, the reduction of immunosuppression by the combinatorial treatment could enable the intrinsically high amount of tumor-specific effector lymphocytes in lymph nodes to elicit the observed antitumor efficacy.

Some advantages of this study are the systematic selection of the combinatorial treatment and the comparison of its therapeutic effect in immunologically different tumor models. These two well-characterized tumor models were used to mimic the expected diversity of the developed tumor cell-based vaccine. Furthermore, this study introduces a novel radio-immunotherapeutic approach, whereby the immunotherapy is a therapeutic vaccine comprising an array of intrinsic tumor antigens combined with a safe non-viral gene therapy to deliver the vaccine adjuvant. The study also has potential limitations. Due to the aggressive nature (fast growth, prone to ulceration) of the B16F10 tumor model, the immune response after 6 days could not be analyzed for this tumor model. Therefore, the comparison between the two immunologically different tumor models could not be performed at later time points. Additionally, this study compares only two tumor models as the vaccine has not yet been developed and tested in other tumor models with different immune profiles to further confirm the tumor immunogenicity-dependent therapeutic response.

In conclusion, the tumor cell-based vaccine using IL-12 GET as an immunological adjuvant is a new and viable immunotherapeutic approach in combination with IR that still needs refinement for translation into the clinic. Nevertheless, this study demonstrates that our developed vaccine can elicit an immune response distantly from the IR tumor, contributing to the response of tumor IR. This interaction was multifaceted and more expressed in less immunogenic B16F10 than more immunogenic CT26 tumor. Therefore, biomarkers to determine tumor immunogenicity and consequently the response to the combinatorial treatment are needed. Further studies into these interactions would be beneficial when designing future treatment regimens.

## Data availability statement

The raw data supporting the conclusions of this article will be made available by the authors, without undue reservation.

## Ethics statement

The animal study was reviewed and approved by Ministry of Agriculture, Forestry, and Food of the Republic of Slovenia, permission no. U34401–1/2015/43 and U34401-35/2020/.

## Author contributions

Conceptualization and supervision - GS and UK. Methodology – GS, UK and TR. Investigation and data collection – TR and KL. Data analysis – TR, KL, UL-T, and KV. Funding acquisition – GS. Data curation – TR, UK, and KL. Writing-original draft preparation – TR. Writing-review and editing - UK, KL, UL-T, KV, AC and GS. All authors contributed to the article and approved the submitted version.

## Funding

This research was funded by the SLOVENIAN RESEARCH AGENCY, grant number P3-0003. The investment was co-financed by THE REPUBLIC OF SLOVENIA and THE EUROPEAN REGIONAL DEVELOPMENT FUND within the scope of SmartGene.si.

## Acknowledgments

We would like to thank Maja Ota (Institute of Oncology Ljubljana, Ljubljana, Slovenia) for her help with preparing histological samples and Mira Lavric (Institute of Oncology Ljubljana, Ljubljana, Slovenia) for her technical help.

## Conflict of interest

The authors declare that the research was conducted in the absence of any commercial or financial relationships that could be construed as a potential conflict of interest.

## Publisher’s note

All claims expressed in this article are solely those of the authors and do not necessarily represent those of their affiliated organizations, or those of the publisher, the editors and the reviewers. Any product that may be evaluated in this article, or claim that may be made by its manufacturer, is not guaranteed or endorsed by the publisher.

## References

[1] Jiao R Allen KJH Malo ME Rickles D Dadachova E. Evaluating the combination of radioimmunotherapy and immunotherapy in a melanoma mouse model. Int J Mol Sci (2020) 21:773. doi: 10.3390/ijms21030773 PMC7037880 31991626

[2] Lumniczky K Candéias SM Gaipl US Frey B. Editorial: Radiation and the immune system: Current knowledge and future perspectives. Front Immunol (2018) 8:1933. doi: 10.3389/fimmu.2017.01933 29410662 PMC5787080

[3] Daguenet E Louati S Wozny A-S Vial N Gras M Guy J-B. Radiation-induced bystander and abscopal effects: important lessons from preclinical models. Br J Cancer (2020) 123:339–48. doi: 10.1038/s41416-020-0942-3 PMC7403362 32581341

[4] Golden EB Frances D Pellicciotta I Demaria S Helen Barcellos-Hoff M Formenti SC. Radiation fosters dose-dependent and chemotherapy-induced immunogenic cell death. Oncoimmunology (2014) 3:e28518. doi: 10.4161/onci.28518 25071979 PMC4106151

[5] Wennerberg E Vanpouille-Box C Bornstein S Yamazaki T Demaria S Galluzzi L. Immune recognition of irradiated cancer cells. Immunol Rev (2017) 280:220–30. doi: 10.1111/imr.12568 PMC5659195 29027232

[6] Santin AD Hermonat PL Hiserodt JC Chiriva-Internati M Woodliff J Theus JW. Effects of irradiation on the expression of major histocompatibility complex class I antigen and adhesion costimulation molecules ICAM-1 in human cervical cancer. Int J Radiat Oncol (1997) 39:737–42. doi: 10.1016/S0360-3016(97)00372-6 9336157

[7] Reits EA Hodge JW Herberts CA Groothuis TA Chakraborty M K.Wansley E. Radiation modulates the peptide repertoire, enhances MHC class I expression, and induces successful antitumor immunotherapy. J Exp Med (2006) 203:1259–71. doi: 10.1084/jem.20052494 PMC3212727 16636135

[8] Grassberger C G Ellsworth S Wilks M Q Keane F K S. Loeffler J. Assessing the interactions between radiotherapy and antitumour immunity. Nat Rev Clin Oncol (2019) 16:729–45. doi: 10.1038/s41571-019-0238-9 PMC13492067 31243334

[9] Zhang F Zheng Z Barman AK Wang Z Wang L Zeng W. Optimal combination treatment regimens of vaccine and radiotherapy augment tumor-bearing host immunity. Commun Biol (2021) 4:78. doi: 10.1038/s42003-020-01598-6 33469123 PMC7815836

[10] Springett GM. Novel pancreatic cancer vaccines could unleash the army within. Cancer Control (2014) 21:242–6. doi: 10.1177/107327481402100311 24955709

[11] McCormick KA Coveler AL Rossi GR Vahanian NN Link C Chiorean EG. Pancreatic cancer: Update on immunotherapies and algenpantucel-l. Hum Vaccines Immunother (2016) 12:563–75. doi: 10.1080/21645515.2015.1093264 PMC4964650 26619245

[12] Curry C.OMMAJr. WT Gorrepati R Piesche M Sasada T Agarwalla P Jones PS. Vaccination with irradiated autologous tumor cells mixed with irradiated GM-K562 cells stimulates antitumor immunity and T lymphocyte activation in patients with recurrent malignant glioma. Clin Cancer Res (2016) 22:2885–96. doi: 10.1158/1078-0432.ccr-15-2163 PMC4911283 26873960

[13] Remic T Sersa G Ursic K Cemazar M Kamensek U. Development of tumor cell-based vaccine with IL-12 gene electrotransfer as adjuvant. Vaccines (2020) 8:111. doi: 10.3390/vaccines8010111 PMC7157224 32121641

[14] Soares KC Rucki AA Wu AA Olino K Xiao Q Chai Y. PD-1/PD-L1 blockade together with vaccine therapy facilitates effector T-cell infiltration into pancreatic tumors. J Immunother (2015) 38:1–11. doi: 10.1097/CJI.0000000000000062 25415283 PMC4258151

[15] Nguyen KG Vrabel MR Mantooth SM Hopkins JJ Wagner ES Gabaldon TA. Localized interleukin-12 for cancer immunotherapy. Front Immunol (2020) 11:575597. doi: 10.3389/fimmu.2020.575597 33178203 PMC7593768

[16] Pavlin D Cemazar M Sersa G Tozon N. IL-12 based gene therapy in veterinary medicine. J Transl Med (2012) 10(1):234. doi: 10.1186/1479-5876-10-234 23171444 PMC3543347

[17] Sersa G Teissie J Cemazar M Signori E Kamensek U Marshall G. Electrochemotherapy of tumors as *in situ* vaccination boosted by immunogene electrotransfer. Cancer Immunol Immunother (2015) 64:1315–27. doi: 10.1007/s00262-015-1724-2 PMC4554735 26067277

[18] Kos S Vanvarenberg K Dolinsek T Cemazar M Jelenc J Préat V. Gene electrotransfer into skin using noninvasive multi-electrode array for vaccination and wound healing. Bioelectrochemistry (2017) 114:33–41. doi: 10.1016/j.bioelechem.2016.12.002 28006672

[19] Kamensek U Tesic N Sersa G Cemazar M. Clinically usable interleukin 12 plasmid without an antibiotic resistance gene: Functionality and toxicity study in murine melanoma model. Cancers (Basel) (2018) 10:60. doi: 10.3390/cancers10030060 PMC5876635 29495490

[20] Ursic K Kos S Kamensek U Cemazar M Miceska S Markelc B. Potentiation of electrochemotherapy effectiveness by immunostimulation with IL-12 gene electrotransfer in mice is dependent on tumor immune status. J Control Release (2021) 332:623–35. doi: 10.1016/j.jconrel.2021.03.009 33705828

[21] Guo S Donate A Basu G Lundberg C Heller L Heller R. Electro-gene transfer to skin using a noninvasive multielectrode array. J Control Release (2011) 151:256–62. doi: 10.1016/j.jconrel.2011.01.014 PMC3101286 21262290

[22] Cuzzubbo S Mangsbo S Nagarajan D Habra K Pockley AG McArdle SEB. Cancer vaccines: Adjuvant potency, importance of age, lifestyle, and treatments. Front Immunol (2021) 11:615240:615240. doi: 10.3389/fimmu.2020.615240 33679703 PMC7927599

[23] Trinchieri G. Interleukin-12: A proinflammatory cytokine with immunoregulatory functions that bridge innate resistance and antigen-specific adaptive immunity. Annu Rev Immunol (1995) 13:251–76. doi: 10.1146/annurev.iy.13.040195.001343 7612223

[24] Lasek W Zagożdżon R Jakobisiak M. Interleukin 12: still a promising candidate for tumor immunotherapy? Cancer Immunol Immunother (2014) 63:419–35. doi: 10.1007/s00262-014-1523-1 PMC3994286 24514955

[25] Yu JW Bhattacharya S Yanamandra N Kilian D Shi H Yadavilli S. Tumor-immune profiling of murine syngeneic tumor models as a framework to guide mechanistic studies and predict therapy response in distinct tumor microenvironments. PloS One (2018) 13:e0206223. doi: 10.1371/journal.pone.0206223 30388137 PMC6214511

[26] Mosely SIS Prime JE Sainson RCA Koopmann J-O Wang DYQ Greenawalt DM. Rational selection of syngeneic preclinical tumor models for immunotherapeutic drug discovery. Cancer Immunol Res (2017) 5:29–41. doi: 10.1158/2326-6066.CIR-16-0114 27923825

[27] Lampreht Tratar U Loiacono L Cemazar M Kamensek U Fazio VM Sersa G. Gene electrotransfer of plasmid-encoding IL-12 recruits the M1 macrophages and antigen-presenting cells inducing the eradication of aggressive B16F10 murine melanoma. Mediators Inflammation (2017) 2017:1–11. doi: 10.1155/2017/5285890 PMC5449735 28596641

[28] Savarin M Kamensek U Cemazar M Heller R Sersa G. Electrotransfer of plasmid DNA radiosensitizes B16F10 tumors through activation of immune response. Radiol Oncol (2017) 51:30–9. doi: 10.1515/raon-2017-0011 PMC5330176 28265230

[29] Schneider CA Rasband WS Eliceiri KW. NIH Image to ImageJ: 25 years of image analysis. Nat Methods (2012) 9:671–5. doi: 10.1038/nmeth.2089 PMC5554542 22930834

[30] Gong Y Sbalzarini IF. Image enhancement by gradient distribution specification. In: Jawahar CV Shan S, editors. Proc. ACCV, 12th Asian conference on computer vision, workshop on emerging topics in image enhancement and restoration. 2014 Nov 1-11. Singapore. Switzerland: Springer (2014). p. w7–p3.

[31] Klein B Loven D Lurie H Rakowsky E Nyska A Levin I. The effect of irradiation on expression of HLA class I antigens in human brain tumors in culture. J Neurosurg (1994) 80:1074–7. doi: 10.3171/jns.1994.80.6.1074 8189262

[32] Lugade AA Moran JP Gerber SA Rose RC Frelinger JG Lord EM. Local radiation therapy of B16 melanoma tumors increases the generation of tumor antigen-specific effector cells that traffic to the tumor. J Immunol (2005) 174:7516–23. doi: 10.4049/jimmunol.174.12.7516 15944250

[33] Vanpouille-Box C Alard A Aryankalayil MJ Sarfraz Y Diamond JM Schneider RJ. DNA Exonuclease Trex1 regulates radiotherapy-induced tumour immunogenicity. Nat Commun (2017) 8:15618. doi: 10.1038/ncomms15618 28598415 PMC5472757

[34] Schaue D Ratikan JA Iwamoto KS McBride WH. Maximizing tumor immunity with fractionated radiation. Int J Radiat Oncol (2012) 83:1306–10. doi: 10.1016/j.ijrobp.2011.09.049 PMC3337972 22208977

[35] Gameiro SR Jammeh ML Wattenberg MM Tsang KY Ferrone S Hodge JW. Radiation-induced immunogenic modulation of tumor enhances antigen processing and calreticulin exposure, resulting in enhanced T-cell killing. Oncotarget (2014) 5:403–16. doi: 10.18632/oncotarget.1719 PMC3964216 24480782

[36] Qiu J Alson D Lee T-H Tsai C-C Yu T-W Chen Y-S. Effect of multiple vaccinations with tumor cell-based vaccine with codon-modified GM-CSF on tumor growth in a mouse model. Cancers (Basel) (2019) 11:368. doi: 10.3390/cancers11030368 PMC6468346 30875953

[37] Kamensek U Ursic K Markelc B Cemazar M Setrajcic Dragos V Sersa G. Mutational burden, MHC-I expression and immune infiltration as limiting factors for *in situ* vaccination by TNFα and IL-12 gene electrotransfer. Bioelectrochemistry (2021) 140:107831. doi: 10.1016/j.bioelechem.2021.107831 33991775

[38] Aiken TJ Komjathy D Rodriguez M Stuckwisch A Feils A Subbotin V. Short-course neoadjuvant *in situ* vaccination for murine melanoma. J Immunother Cancer (2022) 10:e003586. doi: 10.1136/jitc-2021-003586 35039460 PMC8765065

[39] Raj S Bui MM Springett G Conley A Lavilla-Alonso S Zhao X. Long-term clinical responses of neoadjuvant dendritic cell infusions and radiation in soft tissue sarcoma. Sarcoma (2015) 2015:1–8. doi: 10.1155/2015/614736 PMC4735941 26880867

[40] Fisher SA Cleaver A Lakhiani DD Khong A Connor T Wylie B. Neoadjuvant anti-tumor vaccination prior to surgery enhances survival. J Transl Med (2014) 12:245. doi: 10.1186/s12967-014-0245-7 25186961 PMC4156969

[41] Muntjewerff EM Meesters LD van den Bogaart G. Antigen cross-presentation by macrophages. Front Immunol (2020) 11:1276. doi: 10.3389/fimmu.2020.01276 32733446 PMC7360722

[42] Meziani L Deutsch E Mondini M. Macrophages in radiation injury: a new therapeutic target. OncoImmunology (2018) 7:e1494488. doi: 10.1080/2162402X.2018.1494488 30288363 PMC6169587

[43] Gordon S Plüddemann A Martinez Estrada F. Macrophage heterogeneity in tissues: phenotypic diversity and functions. Immunol Rev (2014) 262:36–55. doi: 10.1111/imr.12223 25319326 PMC4231239

[44] Barnes TA Amir E. HYPE or HOPE: The prognostic value of infiltrating immune cells in cancer. Br J Cancer (2017) 117:451–60. doi: 10.1038/bjc.2017.220 PMC5558691 28704840

[45] Teresa Pinto A Laranjeiro Pinto M Patrícia Cardoso A Monteiro C Teixeira Pinto M Filipe Maia A. Ionizing radiation modulates human macrophages towards a pro-inflammatory phenotype preserving their pro-invasive and pro-angiogenic capacities. Sci Rep (2016) 6:18765. doi: 10.1038/srep18765 26735768 PMC4702523

[46] Leblond MM Pérès EA Helaine C Gérault AN Moulin D Anfray C. M2 macrophages are more resistant than M1 macrophages following radiation therapy in the context of glioblastoma. Oncotarget (2017) 8:72597–612. doi: 10.18632/oncotarget.19994 PMC5641155 29069812

[47] Ebert LM MacRaild SE Zanker D Davis ID Cebon J Chen W. A cancer vaccine induces expansion of NY-ESO-1-Specific regulatory T cells in patients with advanced melanoma. PloS One (2012) 7:e48424. doi: 10.1371/journal.pone.0048424 23110239 PMC3482213

[48] Ji D Song C Li Y Xia J Wu Y Jia J. Combination of radiotherapy and suppression of tregs enhances abscopal antitumor effect and inhibits metastasis in rectal cancer. J Immunother Cancer (2020) 8:826. doi: 10.1136/jitc-2020-000826 PMC7592256 33106387

[49] Garnett CT Palena C Chakarborty M Tsang KY Schlom J Hodge JW. Sublethal irradiation of human tumor cells modulates phenotype resulting in enhanced killing by cytotoxic T lymphocytes. Cancer Res (2004) 64:7985–94. doi: 10.1158/0008-5472.CAN-04-1525 15520206

[50] Curiel T. Regulatory T-cell development: is Foxp3 the decider? Nat Med (20072007) 13:250–3. doi: 10.1038/nm0307-250 17342117

[51] Ghaffari-Nazari H Alimohammadi M Alimohammadi R Rostami E Bakhshandeh M Webster TJ. Radiation dose and schedule influence the abscopal effect in a bilateral murine CT26 tumor model. Int Immunopharmacol (2022) 108:108737. doi: 10.1016/j.intimp.2022.108737 35417831

