# Supplementary material for: Tumor cell-based vaccine contributes to local tumor irradiation by eliciting a tumor model-dependent systemic immune response
Source: Front Immunol. 2022 Sep 5;13:974912. doi: 10.3389/fimmu.2022.974912 (PMC9483914; doi:10.3389/fimmu.2022.974912)
Supplement: Supplementary file 1 [file DataSheet_1.docx]

**
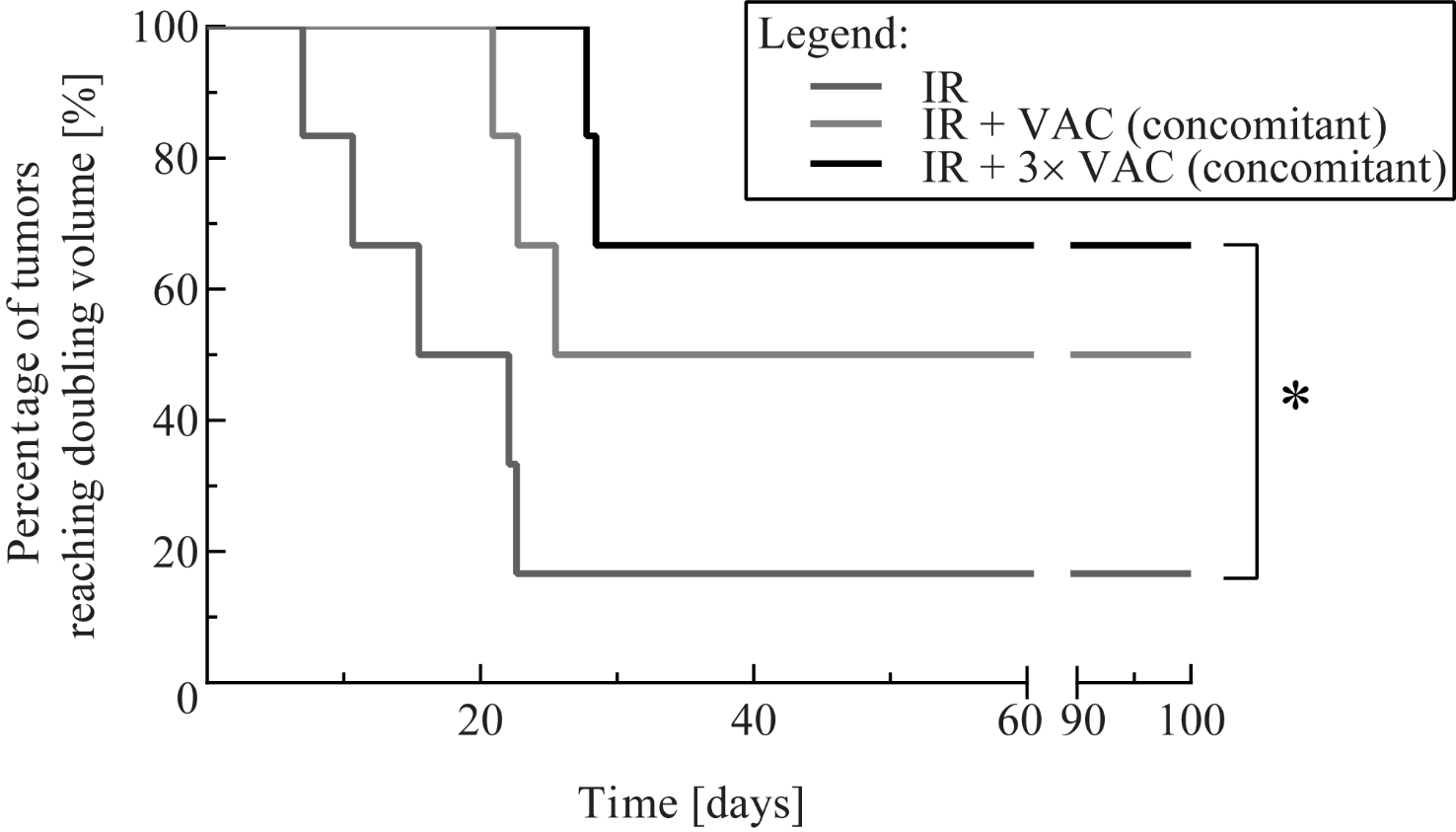
**

**Supplementary Figure 1** **The probability of CT26 tumors reaching doubling volume.** To include censored data (complete responses) the doubling times were compared using log-rank tests. The event, marked as 1, was tumor reaching the doubling volume, while the censored event, marked as 0, was a complete response. The log-rank test for trend confirmed a statistically significant trend, while the log-rank (Mantel-Cox) test showed a significant difference between the IR and the IR + 3× VAC (concomitant) groups, but not between the IR and the IR + VAC (concomitant) groups. **Legend:** * = p < 0.05 between annotated groups, VAC = single-dose vaccination, IR = 5× 5 Gy IR regimen.
